# Free drug percentage of moxidectin declines with increasing concentrations in the serum of marsupials

**DOI:** 10.1016/j.ijppaw.2023.100899

**Published:** 2023-12-27

**Authors:** Eliza K. Stott, Shuai Nie, Nicholas A. Williamson, Lee F. Skerratt

**Affiliations:** a One Health Research Group, Melbourne Veterinary School, Faculty of Science, Werribee, The University of Melbourne, Victoria, Australia; b Melbourne Mass Spectrometry and Proteomics Facility, The University of Melbourne, Victoria, Australia

**Keywords:** Protein binding, Moxidectin, Equilibrium dialysis, LCMS, Wildlife pharmacology, Free drug percentage

## Abstract

Moxidectin (MOX) is a macrocyclic lactone used to eliminate endo and ectoparasites in many mammalian species. It is notably the active ingredient of the anti-parasitic drug Cydectin®, manufactured by Virbac, and is frequently used to treat sarcoptic mange in Australian wildlife. Protein binding plays a significant role in the efficacy of a drug, as the unbound/free drug in plasma ultimately reflects the pharmacologically relevant concentration. This study aimed to investigate the free drug percentage of Moxidectin after *in vitro* spiking into the sera of four sarcoptic mange-susceptible Australian wildlife species; the koala (*Phascolarctos cinereus*), the bare-nosed wombat (*Vombatus ursinus*), the eastern grey kangaroo (*Macropus giganteus*), and the mountain brushtail possum (*Trichosurus cunninghami*). Three concentration points of MOX were tested for each individual: 20 pg/μL, 100 pg/μL and 500 pg/μL. Serum from five individuals of each species underwent an equilibrium dialysis followed by liquid chromatography tandem mass spectrometry (LC-MS/MS). The results showed an atypical concentration dependent binding across all species, where free drug percentage decreased as MOX concentration increased. In addition, wombats showed significantly lower free drug levels. These findings call for further research into the mechanisms of moxidectin protein binding to help understand MOX pharmacokinetics in marsupials.

## Highlights

• The concentration dependent binding of MOX to plasma proteins in serum is atypical in the koala (*Phascolarctos cinereus*), the bare-nosed wombat (*Vombatus ursinus*), the eastern grey kangaroo (*Macropus giganteus*), and the mountain brushtail possum (*Trichosurus cunninghami*).
• Bare-nosed wombats have lower free drug levels of MOX compared to other Australian marsupial species.
• Interspecies differences *in vitro* MOX indicates species specific treatment dosages may need to be developed.
• Further research needs to be conducted to establish the reason behind this novel pattern of protein binding, particularly in bare-nosed wombats.

## Graphical abstract

**Figure:**
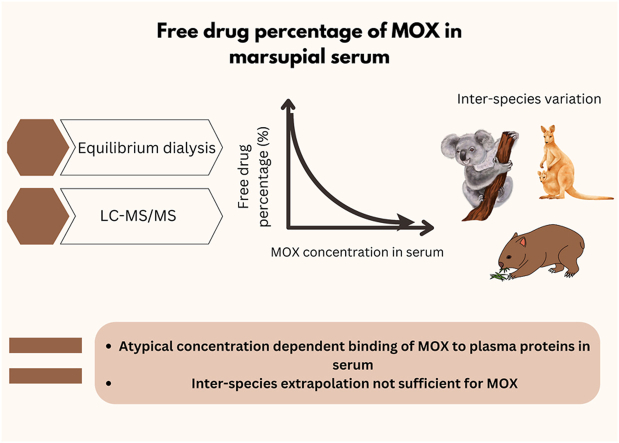

## 1 Introduction

Protein binding in blood can influence the pharmacokinetics of an administered drug, and therefore its clinical effects (Nation et al., 2018). Protein binding affects the bioavailability and distribution of the active drug, as it limits the passage of drugs across biological membranes and barriers (Wanat, 2020). Recognising the important role that protein binding in blood has on the effective drug concentration at the target site is imperative for determining appropriate dosages (Bohnert and Gan, 2013). Moxidectin (MOX) is a macrocyclic lactone (ML) used to eliminate endo and ectoparasites in many mammalian species (Prichard and Geary, 2019). MOX was first used as an injectable formulation for cattle in Argentina in 1989 (Cobb and Boeckh, 2009). Macrocyclic lactones are substrates for P-glycoprotein (P-gp), which is a plasma membrane protein that transports drugs and has significant effects on the excretion of these drugs within the body (Kiki-Mvouka et al., 2010). Unlike some of the other MLs, such as ivermectin, moxidectin has a lower affinity for Pgps (Saunders, 2012). Lespine et al. (2003) first described the major association of MOX to circulating lipoproteins and suggests that the long efficacy of MOX is attributed to this strong association. Bassissi et al. (2004) showed the affinity of MOX to bind to high density lipoproteins, along with highlighting the interspecies differences of the types of lipoproteins that MOX binds to; high density (HDL), low density (LDL), and very low-density (VLDL).

Sarcoptic mange is a parasite of high ecological importance in Australia, as it is responsible for causing significant population decline in Australian wildlife species (Fraser et al., 2016). Sarcoptic mange is caused by infection with the zoonotic astigmatid ectoparasitic mite *Sarcoptes scabiei*, which infests the skin of its host by burrowing into the epidermis (Arlian and Morgan, 2017). Of all Australian wildlife species, bare-nosed wombats are the most seriously affected by this parasite, with sarcoptic mange being one of the main causes of population decline (Fraser et al., 2016). For over 10 years, moxidectin has been used to treat sarcoptic mange Australian wildlife, particularly in wombats, however, there has been very little research conducted on this drug in this species. There is currently a significant amount of debate within the wombat mange treatment community, with treaters regularly using significantly higher dosages of Cydectin® than the current recommendations (Old et al., 2021). There has been one pharmacokinetic trial conducted on four southern hairy-nosed wombats (*Lasiorhinus latifrons*) by Death et al. (2011). This study found that plasma elimination half-life of moxidectin in southern hairy-nosed wombats was shorter, and the peak concentration was higher, compared to MOX in other species (Lanusse and Prichard, 1993; Escudero et al., 1999; Barber et al., 2003).

This study is an investigation into the free drug percentage of MOX, after spiking the drug into serum of four marsupial species affected by sarcoptic mange; the koala (*Phascolarctos cinereus*), the bare-nosed wombat (*Vombatus ursinus*), the eastern grey kangaroo (*Macropus giganteus*), and the mountain brushtail possum (*Trichosurus cunninghami*). Insight into free drug percentage and conversely protein binding affinity of MOX in mange-prone taxa will aid future studies in determining an appropriate dosage of moxidectin that is both safe, and effective, which will in turn have positive outcomes for affected wildlife species.

## 2 Methods

Koala and bare-nosed wombat sera were obtained from healthy animals undergoing routine veterinary procedures in a captive zoological facility and stored in −80 °C conditions at the Melbourne Zoo Veterinary Facility. Eastern grey kangaroo and mountain brushtail possum sera were obtained from free-ranging and overtly healthy individuals in Victoria, that were collected for a previous study. With relevant permits from Zoos Victoria and DEECA, the current study opportunistically utilised leftover sera from a previous study that had been stored in −80 °C conditions for 2 years at The University of Melbourne. Two millilitres of sera were obtained from five individuals from each species: koalas, common wombats, eastern grey kangaroos, and mountain brushtail and common ringtail possums. Sheep sera was used to help establish the method and as a comparative control. This method had not been previously validated using moxidectin, and because wildlife sera is difficult to obtain due to a range of factors, including; ethics, cost, and accessibility, sheep sera was used in validation trials of the extraction process.

Chonker et al. (2019) validated a bioanalytical method of total moxidectin in plasma by Liquid Chromatography Tandem Mass Spectrometry (LC-MS/MS). In 2020, Toh et al. validated a method which couples High Performance Liquid Chromatography Tandem Mass Spectrometry (HPLC-MS/MS) with equilibrium dialysis for quantification of free drug concentration of pazopanib in plasma. This study used both methods to validate a protocol to investigate the free drug percentage of moxidectin in the marsupial species listed.

A Bicinchoninic acid (BCA) assay (Thermo Scientific Pierce BCA Protein Assay Kit) was used to measure total protein concentration in each individual serum sample, to firstly identify any outlier protein levels. Following the guideline of the Food and Drug Administration (FDA) for bioanalytical LCMS assay, a set of six non-zero calibration standards (1.9, 7.8, 31.25, 125, 250, 500, pg/uL) were prepared by adding the appropriate moxidectin working solutions into the respective species’ serum for LCMS assay validation based on linearity, precision and accuracy. The concentrations of MOX for samples were chosen based on the relevant literature, as well as the current guidelines of treating sarcoptic mange in wombats (Bassissi et al., 2004; Hunter et al., 2004; Bassissi et al., 2006; Death et al., 2011; Leathwick and Miller, 2013; Cocquyt et al., 2016; Xiao et al., 2019). The concentrations of MOX included the most field relevant concentration (100pg/uL) as well as lower (20pg/uL and upper (500pg/uL) values. Total and free drug concentrations were determined using the Single-Use Plate Rapid Equilibrium Dialysis (RED) Device (Thermo Fisher Scientific, Rockford, USA) with a molecular weight cut-off of 8000 Da. The fraction unbound (*fu%*) was determined using the following equation: *fu%* = (concentration of analyte in buffer chamber/concentration of analyte in serum chamber) × 100%. Using sheep serum, intra-day precision was measured by relative standard deviation (RSD%), and accuracy (n = 3) at 6 non-zero calibrator levels all revealed acceptable calibration results (accuracy ≤ 15%). Inter-day precisions were not tested as all samples were processed and ran on the same day. The range of concentrations of MOX tested for each species were 20 pg/μL, 100 pg/μL, 500 pg/μL, and each sample was run in triplicates.

LabSolutions Insight LCMS (Shimadzu Scientific, Inc.) was used for data analysis. Statistical analysis was conducted using Microsoft Excel. A single factor ANOVA was performed on each data set to determine if there was an overall significant difference between species, and between concentration points within species. A two tailed *t*-test and a Bonferroni post-hoc test were then conducted on statistically significant results, to establish significant differences between two specific groups (i.e. two species at a given concentration point), and to reduce the possibility of getting a false statistically significant results when testing multiple hypotheses. *p*-values less than 0.05 were considered statistically significant.

## 3 Results

**Fig. 1:**
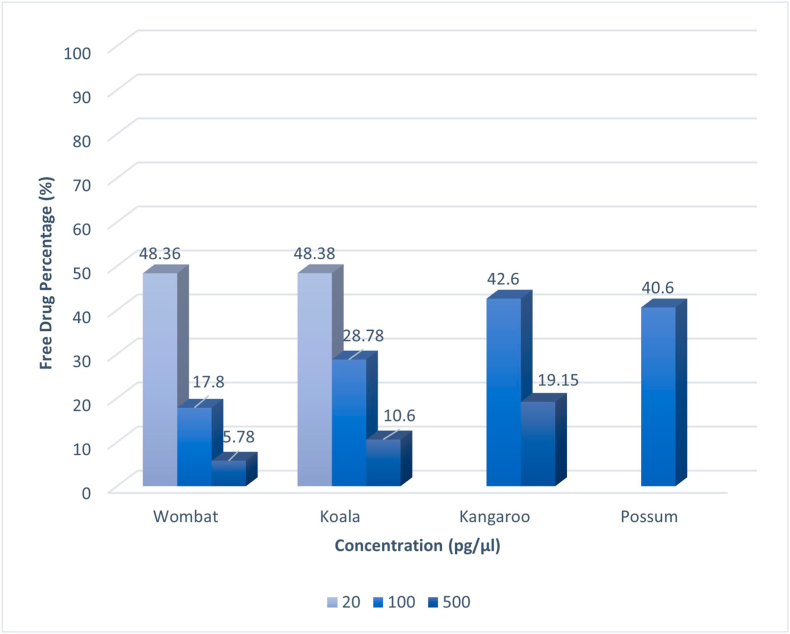
Free drug percentage (%) MOX in serum of koala, kangaroo, and wombat at concentration points 20 pg/μL, 100 pg/μL, and 500 pg/μL. SD; Wombat (20pg/μL- 0.049; 100pg/μL- 0.062; 500 pg/μL), Koala (20pg/μL- 0.099; 100pg/μL- 0.223; 500pg/μL- 0.038),Kangaroo (100pg/μL- 0.053; 500pg/μL- 0.0049), Possum (100pg/μL- 0.042).

A decreasing trend was evident in free drug percentage across all species as MOX concentration increased from 20 pg/μL to 500 pg/μL (Fig. 1; 48.36, 17.8, 5.78 wombat; 48.38, 28.78, 10.6 koala; 42.6, 19.15 kangaroo). Possum and kangaroo had limited sera and therefore not all three concentration points were tested for these two species. The free moxidectin percentages in plasma have statistically significant differences between species at concentration points 100 pg/μL (Fig. 1; ANOVA: p = 0.024, koala, possum, wombat, kangaroo), and 500 pg/μL (ANOVA: p = 1.8 × 10^−3^ between kangaroo, koala, wombat). However, there were not statistically different species differences at 20 pg/μL (koala and wombat). There was also a statistically significant differences between concentration points within each species investigated (ANOVA: wombat p = 2.14 × 10^−8^; koala, p = 7.5 × 10^−5^, kangaroo p = 2.03 × 10^−3^). The average protein concentrations in the serum were; kangaroo (82.78 mg/ml), koala (60.03 mg/ml), possum (84.20 mg/ml), wombat (98.28 mg/ml), and sheep (78.58 mg/ml).

## 4 Discussion

Serum differs from plasma, as it does not contain fibrinogen and other clotting factors. Studies into the *in vivo* binding of drugs, usually only can investigate plasma, whereas *in vitro* studies often use protein isolated from serum (Zeitlinger et al., 2011). Many studies have shown that in most cases, serum and plasma concentrations of analytes are the same, therefore this study, which uses serum, still reveals the pharmacological relevance of the drug in plasma. The results from this study show that free drug percentage decreases as MOX concentration increases and this was consistent across all species tested in this study (Fig. 1). To the best of the authors’ knowledge, the method validated by Toh et al. (2020) has not yet been validated using MOX, therefore this study is a first for this kind of analysis using this anti-parasitic drug. There is currently no available data comparing different *in vitro* free drug concentration levels in MOX. However, in most known cases of protein-drug binding, free drug percentage is fairly constant throughout a clinically relevant range of individuals (Nation et al., 2018).

The atypical pattern of increased protein binding as drug concentrations increase has been described previously, being largely attributed to the drug changing the conformation of the protein and enhancing drug binding to another site on the protein (Levy and Nagashima, 1969; Altmayer, 1995; Berezhkovskiy, 2010). This concept has been well studied in albumin, which undergoes structural changes after ligand binding, where the conformation changes of the protein due to exogenous or endogenous binding can either increase or decrease drug-binding capacity (Sudlow et al., 1975).

MOX is only slightly soluble in water (0.51 mg/L), however, has a higher solubility in hydrophobic fat or lipids (Cobb and Boeckh, 2009; Medicines Development for Global Health, 2018). Due to the large amount of lipids in serum, and the hydrophobic nature of MOX, it is possible that increasing concentration of MOX in serum results in a larger portion of MOX enriched around the protein-bound lipid. The consequences of atypical concentration-dependent protein binding are extremely complex, and largely depends on the factor causing it for the particular drug, which makes the transferability of these *in vitro* studies to *in vivo* outcomes difficult. Deitchman et al. (2018) investigated a case of atypical nonlinear plasma protein binding, where they propose that a failure in testing a wider range of concentration values led to poor predictability of the dosage regimen of tigecycline to treat infections. Therefore, it is recommended for future investigations into free drug concentration that a wide range of concentration points are tested, to reveal the atypical protein binding trends in drugs, which may be missed if only a narrow concentration range are tested.

The statistically significant differences in free drug percentage between the species chosen are noteworthy. While no studies investigating free drug percentage have been done using MOX, there have been reports of inter-species variations in the degree of binding to a range of other drugs because of differences in affinity and capacity (Belpaire, 1986). A study conducted by Nouri-Sorkhabi et al. (1996) reported the total phospholipid (PL) of plasma from marsupials comparable with other species, however, states that the bare-nosed wombat is an exception, which had values much lower. Phospholipids can enhance the bioavailability of low aqueous solubility, such as moxidectin, and these lower levels of PLs reported in wombats may contribute to some of the lower MOX bioavailability found in this study (Singh et al., 2017). Our study showed that wombats had lower levels of MOX free drug percentage compared with the other three species, which may have therapeutic consequences when treating wombats with sarcoptic mange (Fig. 1). However, further research needs to be conducted to determine this, particularly in pharmacokinetics and field efficacy trials with the bare-nosed wombat. It is also important to note that wombats had a higher average protein concentration compared to the other animals in the study, which may add to the possible higher degree of drug protein binding, and thus lower free drug percentages.

The effect that disease has on protein binding has been widely reported in the literature, and changes in protein levels have been observed in sarcoptic mange in other species (Tillement et al., 1978). Acute phase proteins have been reported to increase with severity of sarcoptic mange in Iberian ibex (*Capra pyrenaica pyrenaica*) (Rahman et al., 2010) as well as in Capybaras (*Hydrochoerus hydrochaeris*) (Bernal et al., 2011). Skerratt et al. (1999) reported significantly lower total protein and albumin levels in *S.scabiei* infested wombats compared to healthy captive wombats. However, Skerratt (2003) later suggested that these changes only occurred when infestation with *S.scabiei* became chronic. The changes of serum proteins with increasing severity of sarcoptic mange increases may indeed affect the protein binding of MOX. We suggest future research compares the *in vitro* protein binding of MOX in the sera of wombats at varying severities of infestation with sarcoptic mange.

## 5 Conclusion

The novel results found for free drug percentage of MOX in Australian wildlife sera suggest atypical non-linear concentration-dependent protein binding. The statistically significant difference in MOX protein binding between wombats and the other species at varying MOX concentration points highlights that interspecies differences *in vitro* alone call for species specific treatment regimens to be developed.

## Funding

LFS was supported by the Australian Research Council [grant FT190100462].

## Scientific approval

A scientific permit through the Department of Environment, Land, Water, and Planning [ID 10010022] was obtained for the transportation of koala and wombat sera from Melbourne Zoo to The University of Melbourne. Approval for the use of the sera was also obtained through Zoos Victoria [ZV21008].

## Declaration of competing interest

The authors declare no conflict of interest.

## Figures and Tables

**Table:** 

NOMINAL CONCENTRATION (PG/μL)	INTRA-DAY ACCURACY (%)	%RSD
**2**	110.51	2
**8**	96.28	4.48
**31**	91.57	3.61
**125**	99.29	2.1
**250**	103.23	6.2
**500**	99.103	10.65

**Table:** 

Nominal concentration (pg /μl)	INTRA-DAY ACCURACY (%)	%RSD
2	124.46	29.93
8	85.6	2.6
31	91.093	7.34
125	102.33	10.92
250	92.837	5.1
500	103.687	11.34

**Table:** 

Nominal concentration (pg /μl)	INTRA-DAY ACCURACY (%)	%RSD
2	65.31	87.19
8	86.007	10.08
31	77.013	17.54
125	99.64	0.98
250	99.8	2.75
500	101.707	458

**Table:** 

Nominal concentration (pg /μl)	INTRA-DAY ACCURACY (%)	%RSD
2	123.53	4.25
8	98.577	7.77
31	82.67	1.72
125	94.33	4.94
250	96.913	11.48
500	103.973	16.09

**Fig. A.2.1:**
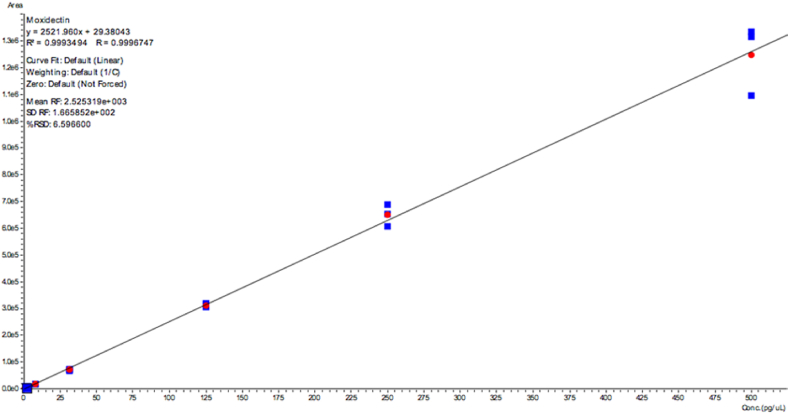
Kangaroo serum validation curve.

**Fig. A2.2:**
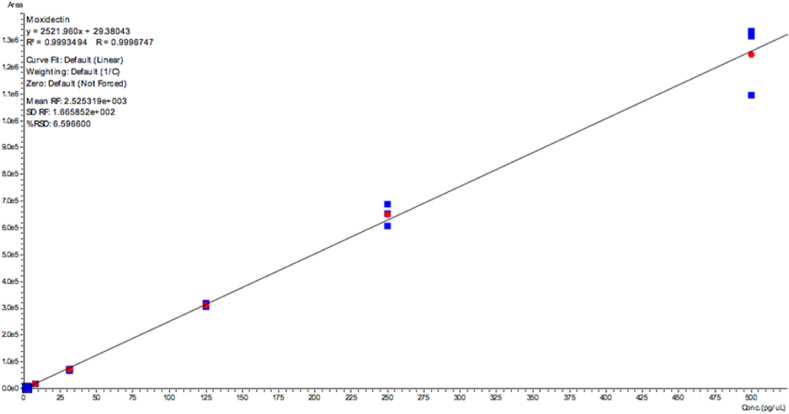
Koala serum validation curve.

**Fig. A2.3:**
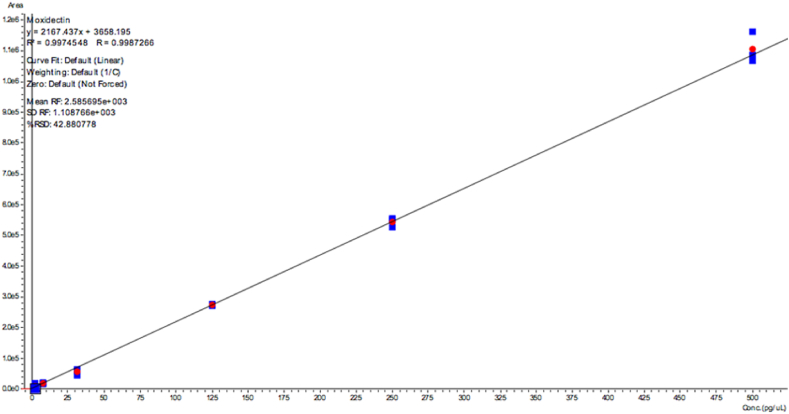
Possum serum validation curve.

**Fig. A2.4:**
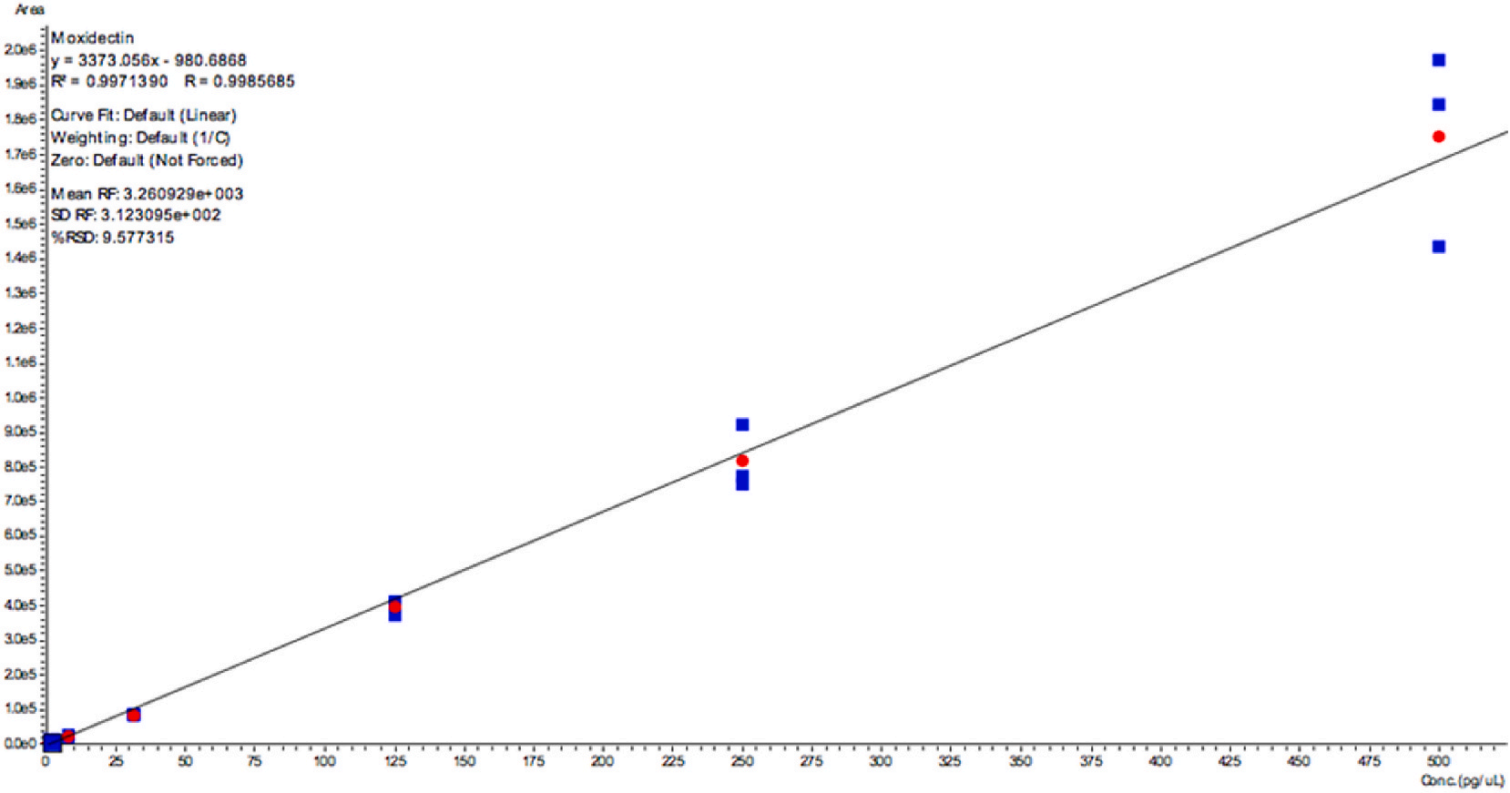
Wombat serum validation curve.

**Table A2:** Concentrations of MOX tested in each species

Species	Concentrations MOX tested
Koala	20 pg/μl, 100 pg/μl, 500 pg/μl
Wombat	20 pg/μl, 100 pg/μl, 500 pg/μl
Kangaroo	100 pg/μl, 500 pg/μl
Possum	100 pg/μl

## Appendix

### A3: Detailed methods

#### A3.1 Sample collection

Serum differs from plasma, as it does not contain fibrinogen and other clotting factors. Studies into the *in vivo* binding of drugs, usually only can investigate plasma, whereas *in vitro* studies often use protein isolated from serum (Zeitlinger et al., 2011). Many studies have shown that in most cases, serum and plasma concentrations of analytes are the same, therefore this study, which uses serum, will still reveal the pharmacological relevance of the drug in plasma. Samples were obtained from sera previously extracted from animals undergoing veterinary procedures. Two millilitres of serum were obtained from five koalas (*Phascolarctos cinereus*), common wombats (*Vombatus ursinus*), eastern grey kangaroos (*Macropus giganteus*) and mountain brushtail possums (*Trichosurus cunninghami*). Sheep serum was used to help establish the method and as a comparative control. This method had not been previously validated using moxidectin, and because wildlife serum is difficult to obtain due to a range of factors, including; ethics, cost, and accessibility, sheep serum was used in validation trials of the extraction process. However, both sheep and wildlife serum were used in the validation of the LCMS assay. The eastern grey kangaroo, mountain brushtail possum, and sheep sera were sourced from and stored at the Melbourne Veterinary School in −80 °C conditions. The wombat and koala sera were sourced from and stored at the Melbourne Zoo Veterinary Facility in −80 °C conditions. A scientific permit [ID 10010022] was obtained for the transportation of koala and wombat sera from the Melbourne Zoo to The University of Melbourne through the Department of Environment, Land, Water, and Planning.

#### A3.2 Evaluation of serum protein concentration

A Bicinchoninic acid (BCA) ssay (Thermo Scientific Pierce BCA Protein Assay Kit) to measure total protein concentration was conducted on each individual serum sample, to firstly identify any outlier protein levels. Samples were diluted 10 fold using water. 25 μL of each bovine serum albumin (BSA) standard with varying concentrations or diluted unknown samples were added to a 96 well plate. 200 μL of the BCA assay working reagent was added to each well. The plate was covered and incubated at 37 °C for 30 min, before being cooled to room temperature. The absorbance was measured at 562 nm on a plate reader. An external calibration curve was produced based on the BSA standards and were used to determine the protein concentration of each unknown sample.

#### A3.3 LCMS

The LC-MS/MS setup consisted of a high-performance liquid chromatography (HPLC) system coupled to a triple quadruple mass spectrometer (Shimadzu LCMS-8050, Shimadzu Scientific, Inc.). Chromatographic separation of moxidectin and d3-moxidectin as an internal standard was carried out using an Agilent RRHD Eclipse C18 Column (2.1 × 100 mm, 1.8 μm). The injection volume used was 10 μL and total chromatographic run time was 15.0 min. The mobile phase used consisted of 0.1% v/v acetic acid in water/acetonitrile/methanol = 2/4/4 (v/v/v) for isocratic gradient. Quantification of all analytes was more readily achieved with improved sensitivity in negative multiple reaction monitoring (MRM) mode. Within the electrospray ionization source, the nebulizer gas was set to 2.0 L/min, heating gas to 10 L/min, drying gas to 10 L/min, interface temperature to 375 °C, desolvation line temperature to 250 °C, heat block temperature to 400 °C and interface voltage to 4000 V. The MRM precursor ion → product ion transitions for MOX and d3-MOX IS were m/z 638.40 → 236.30 and m/z 641.3 → 239.3, respectively.

#### A3.4 Preparation of stock solutions, calibration standards and quality controls for validation

Stock solutions containing moxidectin (1ug/ul) were prepared in methanol (MeOH) and stored at −20 °C. Working solutions were serially diluted down from stock solutions using methanol (MeOH). Following the guideline of FDA for LCMS assay, a set of six non-zero calibration standards (1.9, 7.8, 31.25, 125, 250, 500, pg/uL) were prepared by adding the appropriate moxidectin working solutions into the respective species’ serum for LCMS assay validation based on linearity, relative standard deviation (RSD) and accuracy. The concentrations of MOX for samples were chosen based on the relevance in the current literature around treatment doses (20 pg/μL, 100 pg/μL, 500 pg/μL). However, due to limitations in volume of wildlife serum, not all of the concentration points were tested. For these species, concentration points were chosen based on the most pharmacologically relevant concentration:

#### A3.4 Preparation of plasma samples for equilibrium dialysis

The collected serum samples were subsequently analysed for their total and free drug concentrations using the following method: Single-Use Plate Rapid Equilibrium Dialysis (RED) Device (Thermo Fisher Scientific, Rockford, USA) with a molecular weight cut-off of 8000 Da was used. 15 μl of respective MOX concentrations (20 pg/μl, 100 pg/μl, 500 pg/μl) were added into respective Eppendorf tubes, and dried down using a SpeedVac vacuum concentrator for 5 min 150 μl of respective serum was added into each Eppendorf tube. A total of 100 μL of the serum spiked with MOX and 350 μL of Phosphate Buffered Saline (PBS) were loaded into the sample chamber and buffer chamber respectively, according to manufacturer's instructions. In the validation stage, both 50 μl serum/300 μl PBS, and 100 μl serum/350 μl PBS were trialed, and 100 μl serum/350 μL PBS produced better reproducibility. The samples were then incubated at 37 °C on an orbital shaker (LM-570D, Yihder Technology, Xinbei, China) at 200 rpm for 6 h to establish equilibrium. In the validation stages, both a 4 h and 6-h dialysis time were trialed, with 6 h having better reproducibility. Content from both sample and buffer chambers was collected after equilibrium dialysis.

#### A3.5 Post-dialysis preparation of samples for LCMS analysis

25 μL of the respective serum sample or PBS sample from the dialysis was pipetted to a 2 mL Eppendorf tube. 25 μl of PBS was added to the samples of serum spiked with moxidectin. 25 μl of respective drug free serum was added to PBS samples. 175 μL of MeOH and 25 μL of internal standard (d3 MOX at 100 pg/μl) in methanol were added into each Eppendorf tube. The tube was vortex-mixed for 30 s, followed by centrifugation at 14,000×*g* for 10 min. 150 μl of the upper layer was transferred to separate glass vials for LCMS analysis.

The fraction unbound (*fu%*) was determined using the following equation: *fu%* = (concentration of analyte in buffer chamber/concentration of analyte in serumhamber) × 100%

#### A3.6 Data analysis

LabSolutions Insight LCMS (Shimadzu Scientific, Inc.) was used for data analysis. Each sample used as calibration standards were run in triplicates. The least-squares linear regression analysis was employed to plot the calibration curves. The validation of the method was carried out following guidelines for Bioanalytical Method Validation published by the FDA for intra-day precision and accuracy. Statistical analysis was conducted using Microsoft Excel. A single factor ANOVA was performed on each data set to determine if there was an overall significant difference between species, and between concentration points within species. A two tailed *t*-test and a Bonferroni post-hoc test were then conducted on statistically significant results, to establish significant differences between two specific groups (i.e. two species at a given concentration point), and to reduce the possibility of getting a statistically significant results when testing multiple hypotheses. *p*-values less than 0.05 were considered statistically significant.
